# The Importance of Autoantibody Detection in Autoimmune Hepatitis

**DOI:** 10.3389/fimmu.2015.00222

**Published:** 2015-05-13

**Authors:** Eduardo Luiz Rachid Cancado, Clarice Pires Abrantes-Lemos, Debora Raquel B. Terrabuio

**Affiliations:** ^1^Department of Gastroenterology, Clinical Gastroenterology and Clinical Hepatology of Hospital das Clinicas, University of São Paulo School of Medicine, São Paulo, Brazil; ^2^Laboratory of Immunopathology of Schistosomiasis, Institute of Tropical Medicine, University of São Paulo, São Paulo, Brazil; ^3^Laboratory of Tropical Gastroenterology and Hepatology, Institute of Tropical Medicine, São Paulo, Brazil

**Keywords:** autoimmune hepatitis, autoimmune liver diseases, liver autoantibodies, antinuclear antibodies, antismooth muscle antibodies, anti-LKM1 antibodies, anti-SepSecS

The autoantibodies in autoimmune hepatitis (AIH) are extensively used for its diagnosis and classification. However, they are also useful for defining the prognosis and inferring clinical behavior.

## For the Diagnosis and Classification of AIH

In accordance with the classical criteria for the diagnosis of AIH, the importance of autoantibody testing is diluted among 12 parameters (1). Only adult patients with autoantibody reactivity greater than 1/80 for the classical markers, such as anti-smooth muscle antibodies (ASMAs), anti-nuclear antibodies (ANAs), and anti-liver/kidney microsome type 1 (anti-LKM1) antibodies, score three points. When these markers are absent, other secondary antibodies, such as anti-soluble liver/pancreas antigen (anti-SLA/LP) and liver cytosol type 1 (anti-LC1), should be tested. It is possible to reach a definite diagnosis of AIH without any autoantibody reactivity, and this occurs in approximately 5–10% of all AIH cases.

Further, a simplified scoring system was proposed based on only four parameters: absence of viral hepatitis antibodies, increased IgG levels, typical histological features, and reactivity for liver autoantibodies (each with a maximum score of two points under standard specifications) (2). As stated in the interpretation of these simplified criteria, it is impossible to have a definite diagnosis without any reactivity for autoantibodies, and we know this is not true.

The reactivity of autoantibodies in AIH is crucial for its classification. ASMAs and ANAs are markers of type 1 AIH (AIH-1), and anti-LKM1 and anti-LC1 antibodies are markers of type 2 AIH (AIH-2). Anti-SLA/LP antibodies were initially considered markers of a third type of AIH, but this subject is still under debate. The major autoantibodies, their corresponding target antigens, the techniques for their detection, and the main features of AIH that they are the markers are displayed in Table 1.

**Table 1:** **Main autoantibody markers of AIH with their corresponding target antigens, techniques of detection, and AIH clinical features (1, 3–8)**.

Autoantibodies	Target antigen	Techniques of detection	AIH clinical features
Anti-smooth muscle antibodies (ASMA)	Filamentous actin	IIF – rodent stomach and kidney sections – reaction on stomach muscular layers, vessels, glomeruli, and fibrils of tubular cells (tubular pattern)	Ratio female: male – 4:1 Higher levels of γglobulins
AIH-1 (70%); frequently associated with anti-nuclear antibodies			
The most common marker of AIH in all ages			HLA susceptibility DR3 and, North and South America countries with DR13
Anti-actin antibodies	Filamentous actin	IIF – cell culture (human fibroblasts, HEp2 cells)	
		ELISA (less specific); high reactivity in other liver diseases and even without ASMA reactivity	
Anti-nuclear antibodies (ANA)	Histone, Ro (SSA)	IIF (homogeneous and speckled patterns)	Isolated ANA are more common in adults
50–70% of patients with AIH-1, mainly in association with ASMA		Other patterns (nucleolar, centromere, nuclear dots, and nuclear envelope) are not related to AIH	Markers of a less aggressive disease Higher association with rheumatologic diseases
		ELISA (anti-histone and anti-Ro antibodies)	
			Relationship with HLA DR4; in Brazil there is no relationship between ANA reactivity and HLA DR
Anti-liver kidney microsome antibodies type 1 (anti-LKM1) 15% of patients with AIH 90% of patients with AIH-2	Cytochrome CYPIID6	IIF – liver and kidney tissue sections – homogeneous fluorescence in hepatocytes, and reactivity in proximal renal tubular cells	AIH-2 More frequently detected in young children, even younger than 5 years old; less commonly in patients older 20 years of age
		Immunoblotting (mainly 50, 56, and 66 kDa)	Acute liver failure
		Other techniques: immunodiffusion, ELISA, LIA	
			Relationship with class II HLA DR7 and DQ2 (Brazil and Canada); DR3 (Western Europe)
			Relapses more frequent
Anti-liver cytosol type 1	Formiminotransferase cyclodeaminase	IIF (when anti-LKM1 antibodies are negative)	Few studies with patients carrying these antibodies without anti-LKM1 More severe and less responsive to treatment forms of AIH
30–40% of patients with AIH-2; only 10% of AIH-2 patients with these antibodies alone		Homogenous reactivity in hepatocytes, with fading fluorescence reactivity around centrilobular venules; no reactivity in proximal tubules	
More frequently in association with anti-LKM1		Immunoblotting: 62 kDa with liver antigen sources	
		Other techniques: immunodiffusion, ELISA	
Anti-soluble liver/liver pancreas antibodies (anti-SLA/LP) One-third of patients with AIH without the classical markers	Sep (O-phosphoserine) tRNA: Sec (selenocysteine) tRNA synthase; (SepSecS → anti-SepSecS)	ELISA, immunoblotting, line immunoassay No reactivity by IIF	More relapses after treatment withdrawal 90% reactivity together with anti-RO 52 antibodies) High association with HLA DR3 Higher levels of γglobulins
More frequently detected in AIH-1 than AIH-2			
15–20% of all AIH patients			

*AIH, type 1 autoimmune hepatitis; AIH-2, type 2 autoimmune hepatitis; ASMA, anti-smooth muscle antibodies; ANA, antinuclear antibodies; anti-LKM1, anti-liver kidney microsome antibodies type 1; Anti-SLA/LP: anti-soluble liver/liver pancreas antibodies; IFI: indirect immunofluorescence*.

### Anti-smooth muscle antibodies/antimicrofilament antibodies/anti-actin antibodies

**Figure 1:**
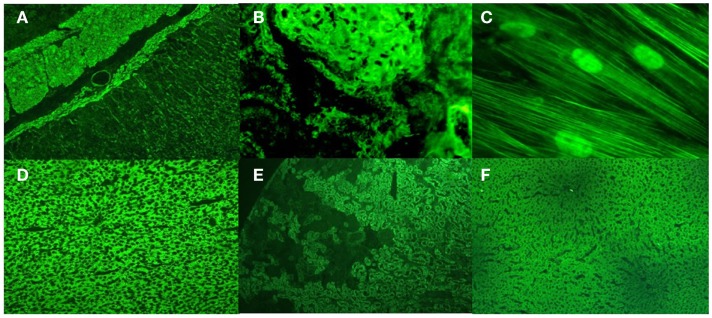
**Upper panel**: Anti-smooth muscle and anti-microfilaments antibodies in type 1 autoimmune hepatitis. **(A)** ASMA in rat stomach sections; **(B)** the tubular pattern of ASMA in rat kidney sections; **(C)** anti-microfilament and antinuclear antibodies in human fibroblasts. **Lower panel**: Anti-liver kidney microsome antibodies type 1 (anti-LKM1) and anti-liver cytosol antibodies type 1 (anti-LC1) in type 2 autoimmune hepatitis; **(D)** anti-LKM1 in rat liver sections; **(E)** anti-LKM1 in rat kidney sections; **(F)** anti-LC1 in rat liver sections.

Anti-smooth muscle antibodies were initially described in rodent stomach sections in 1965, with reactivity in the *Muscularis mucosae*, the submucosa vessel walls, and the muscular layers. However, the patterns observed in kidney sections yield more information because the target antigen for ASMAs is the filamentous form of actin (F-actin), and this information is provided via the presence of a tubular pattern (a fluorescent reaction in the vessels, glomeruli and fibrils inside the tubular cells in unfixed rodent kidney sections, Figures 1A,B) (9).

To properly detect anti-F-actin antibodies by indirect immunofluorescence, it is necessary to identify the reactivity of microfilaments in cell culture (Figure 1C). To better visualize the microfilaments in cells in lower dilutions, one must heat inactivate the serum samples at 56°C for 30 min or dilute them in calcium chelating solutions to inactivate a serum labile and calcium-dependent protein that severs F-actin from the substrate, called gelsolin (10).

The vascular and glomerular patterns of ASMAs are frequently seen in other clinical conditions and frequently in lower titers. The antigenic specificity in this situation usually is for other components of the cytoskeleton, such as vimentin, desmin, tubulin, or cytokeratin. These anti-cytoskeleton autoantibodies are more easily observed when the cells are treated with vinblastine or colchicine. Sometimes, it is important to choose a specific type of cell culture because the intermediate filaments are not the same for all cell types. International diagnostic scoring systems make no specification for ASMA patterns, and a vascular/stomach pattern higher than 1/80 has the same meaning of a tubular pattern higher than 1/320, which is not definitely true.

There is a commercially available anti-F-actin ELISA, and we tested this assay in different patient groups with liver diseases. The AIH-1 group in which only patients with ASMAs with the tubular pattern and with F-actin specificity were included had the highest reactivity frequency. Almost, all patients demonstrated reactivity according to this assay. However, the control groups also demonstrated a high reactivity frequency, even when the cut off was shifted up 4 SDs, which differed from the manufacturer’s recommendations. Furthermore, even patients without reactivity to ASMAs had some reactivity according to this assay. Thus, it is uncertain whether this commercial ELISA always detects the same antigen as indirect immunofluorescence. For this reason, although highly related to the tubular pattern of ASMAs (11), we still do not recommend this assay for routine examinations.

### Anti-nuclear antibodies

The two more common patterns of ANAs detected in AIH are the homogeneous and speckled. However, often it turns out to be an association of patterns. Sometimes, the pattern changes during the course of treatment or even during the process of serum titration. When antigenic specificities are tested, histone H1 and SSA/RO are the most prevalent target antigens. Frequently, ANAs are detected together mainly with ASMAs but also with anti-LKM1, anti-LC1, and anti-SLA/LP antibodies, all of them more specific for AIH. In this situation, the type of AIH that the patient should be classified as is not clear.

Although the ANA detection is universally performed using HEp2 cells as substrate, a review of the International Group of AIH suggested HEp2 cells should not be used initially as screening because of the high positivity rate in lower dilutions (less than 1/80), and for children the reactivity in those dilutions is useful in characterizing AIH subtypes (1, 3).

An unresolved issue on the reactivity of the ANA is that there is no specification for patterns in the diagnostic scoring systems. Both international scoring systems for the diagnosis of AIH score points for ANA reactivity in the presence of centromeric, nucleolar, nuclear dots and nuclear envelope patterns, even in the presence of rheumatic diseases that, *per se*, justify their presence. Only homogeneous and speckled patterns should be considered AIH markers.

### Anti-liver kidney microsome antibodies type 1 and anti-liver cytosol antibodies type 1

Anti-LKM1 and anti-LC1 antibodies are the primary markers of AIH-2. Their target antigens are the enzymes cytochrome CYP2D6 and formiminotransferase cyclodeaminase, respectively. Anti-LKM1 antibodies are characterized by reactivity in proximal renal tubules in the renal cortex and by a diffuse reaction in hepatocytes in rat tissue sections. Anti-LC1 antibodies are characterized by reactivity in hepatocytes with a weak fluorescence around the centrilobular veins without any reactivity in renal tubules (Figures 1D–F). Anti-LC1 antibodies are only detected by indirect immunofluorescence if anti-LKM1 antibodies are not present simultaneously. Both antibodies are also determined using purified rat and human liver antigens by immunoblotting and immunodiffusion and by ELISA and line immunoassay using recombinant antigens. By immunoblotting with rat antigens, anti-LKM1 antibodies are depicted in three bands of 50, 56, and 66 kDa. The 50 kDa band is the most important. For anti-LC1, only one 62 kDa band is observed when using human antigens. Anti-LKM1 antibodies are markers in 90% of patients with AIH-2, while anti-LC1 in 25–40% of patients, more frequently in association with anti-LKM1. Anti-LC1 antibodies alone are markers for approximately 10% of patients with AIH-2. Anti-LKM1 antibodies are detected in <5% of patients with chronic hepatitis C; anti-LC1 antibodies are scarcely reactive in this disease, and both are rarely reactive in AIH-1.

### Anti-soluble liver antigen/liver-pancreas antibodies/antiSepSecS antibodies

Anti-SLA/LP antibodies were initially described in 1981 with the name anti-liver pancreas, and in 1987 as anti-SLA antibodies. The target antigen of anti-SLA/LP is Sep (O-phosphoserine) tRNA: Sec (selenocysteine) tRNA synthase (12), named SepSecS according to the Nomenclature Commission of the Human Genome Organization. The enzyme SepSecS catalyzes the last step of selenocysteine synthesis. These antibodies can be detected by ELISA, immunoblotting, radioligand assay, and line immunoassay using purified or recombinant antigens.

In a multicenter study from Germany, USA, Japan, and Brazil, the reactivity of anti-SepSecS was approximately 15% when considering patients with AIH, and was approximately 30% when considering AIH without the classical markers (4). Close to 10% of patients with chronic hepatitis C exhibited these markers. In our institution, anti-SepSecS reactivity was present in 33% of patients with AIH without the classical markers; in 23% of patients with type 1 AIH; and 13% of patients with type 2 AIH. Overall, 22% of 243 patients with AIH were positive compared to only 4% of 151 controls that included patients with chronic hepatitis C with autoimmune features, primary biliary cirrhosis, primary sclerosing cholangitis, celiac disease, and healthy individuals.

In a review of adult and pediatric patients from our hospital, we determined the distribution of AIH subtypes. From 227 patients with AIH, 78% of 177 patients had AIH-1; 47% had ASMAs; 16% had isolated ANAs; and 37% had both markers. Ninety percent of patients with ASMA reactivity demonstrated specificity for anti-microfilament antibodies. Fourteen percent of 32 patients had AIH-2; 75% with isolated anti-LKM1 antibodies; 9% with isolated anti-LC1 antibodies; and 16% with both markers. Hence, 90% of patients with AIH-2 had anti-LKM1 antibodies. Eight percent of the whole cohort of AIH patients demonstrated no reactivity for the classical markers, and one third of them tested positive for anti-SepSecS antibodies (5).

## For Defining the Prognosis or Inferring the Clinical Features and Behavior of AIH

Patients with anti-LKM1 reactivity are usually younger than patients with other autoantibody profiles, and patients with anti-SepSecS or with isolated ANA reactivity tend to be older (4, 6). AIH-2 is usually more aggressive, and acute liver failure is more common in these patients. Patients with AIH-2 exhibited an IgA deficiency more frequently; on the other hand, the levels of gamma globulin and IgG are considerably higher in patients with anti-microfilament and anti-SepSecS antibodies than in patients with anti-LKM1 (4–6).

Patients with anti-LC1, anti-SepSecS, and anti-F-actin antibodies have a worse prognosis than their counterparts with isolated ANAs in which the response to treatment is much better than in patients with other serological markers (13, 14). Those who remain reactive to anti-F-actin greater than 1/40 and to ASMA greater than 1/80 after treatment usually have histological activity (15). Patients with reactivity for anti-SepSecS have relapses more frequently than other patients after treatment withdrawal. However, the prognostic implications ascribed to these antibodies could also be related to anti-Ro52 reactivity due to the almost invariable concomitance of these two types of autoantibodies (7).

One of the explanations for the clinical differences among patients with AIH is related to their genetic background according to MHC class II. In Western Europe and North America, AIH-1 has a primary association with HLA DR3 and a secondary association with HLA DR4. In contrast, HLA DR4 is the primary association in Japan and Mexico. For AIH-2, the primary association is HLA DR3 in Europe and HLA DQ2 and HLA DR7 in Canada. AIH-1 in South America (Argentina, Brazil and Venezuela) is related to HLA DR13, but only in Brazil, this susceptibility was related to anti-microfilament antibody reactivity (8). In Brazilian patients, for AIH-2 the susceptibility was related to HLA DR7, for patients with anti-SepSecS reactivity to HLA DR3 (like in other countries), and for patients with isolated ANA reactivity no specific association with class II alleles was observed.

## Conclusion

In summary, in AIH, there is no need to test the entire panel of autoantibodies. The presence of one is sufficient to facilitate the diagnosis, and the treatment is the same regardless of the serological markers. It is important to highlight that the reactivity of autoantibodies is one of the several important parameters to diagnose AIH, as are the high levels of gamma globulins; the typical histological alterations; the therapeutic response to corticosteroids and azathioprine; and the relapse after discontinuation of treatment. On the other hand, the diagnosis can be made even without any serological markers. However, to deeply understand the patients, at least, five autoantibodies (ASMA, anti-LKM1, anti-LC1, ANA, and anti-SepSecS) should be tested with the hope of explaining the clinical manifestations, biochemical changes, genetic background, clinical course and treatment response and, perhaps, of identifying a triggering agent.

## Conflict of Interest Statement

The authors declare that the research was conducted in the absence of any commercial or financial relationships that could be construed as a potential conflict of interest.
